# Standardized Phylogenetic Classification of Human Respiratory Syncytial Virus below the Subgroup Level

**DOI:** 10.3201/eid3008.240209

**Published:** 2024-08

**Authors:** Stephanie Goya, Christopher Ruis, Richard A. Neher, Adam Meijer, Ammar Aziz, Angie S. Hinrichs, Anne von Gottberg, Cornelius Roemer, Daniel G. Amoako, Dolores Acuña, Jakob McBroome, James R. Otieno, Jinal N. Bhiman, Josie Everatt, Juan C. Muñoz-Escalante, Kaat Ramaekers, Kate Duggan, Lance D. Presser, Laura Urbanska, Marietjie Venter, Nicole Wolter, Teresa C.T. Peret, Vahid Salimi, Varsha Potdar, Vítor Borges, Mariana Viegas

**Affiliations:** University of Washington, Seattle, Washington, USA (S. Goya);; University of Cambridge, Cambridge, UK (C. Ruis); University of Basel and SIB, Basel, Switzerland (R.A. Neher, C. Roemer, L. Urbanska);; National Institute for Public Health and the Environment, Bilthoven, the Netherlands (A. Meijer, L.D. Presser);; World Health Organization Collaborating Centre for Reference and Research on Influenza, Melbourne, Victoria, Australia (A. Aziz);; University of California Santa Cruz, Santa Cruz, California, USA (A.S. Hinrichs, J. McBroome);; National Institute for Communicable Diseases of the National Health Laboratory Service, Johannesburg, South Africa (A. von Gottberg, J.N. Bhiman, J. Everatt, N. Wolter);; University of Witwatersrand, Johannesburg, South Africa (A. von Gottberg, J.N. Bhiman, N. Wolter);; University of KwaZulu-Natal, Durban, South Africa (D.G. Amoako);; Universidad Nacional de La Plata, Buenos Aires, Argentina (D. Acuña, M. Viegas);; National Scientific and Technical Research Council, Buenos Aires, Argentina (D. Acuña, M. Viegas);; Theiagen Genomics, Highlands Ranch, Colorado, USA (J.R. Otieno);; Autonomous University of San Luis Potosí, San Luis Potosí, Mexico (J.C. Muñoz-Escalante);; Rega Institute for Medical Research, Leuven, Belgium (K. Ramaekers);; University of Edinburgh, Edinburgh, Scotland, UK (K. Duggan);; University of Pretoria, Pretoria, South Africa (M. Venter);; University of Texas Medical Branch, Galveston, Texas, USA (T.C.T. Peret);; Tehran University of Medical Sciences, Tehran, Iran (V. Salimi);; ICMR National Institute of Virology, Pune, India (V. Potdar);; National Institute of Health Doutor Ricardo Jorge, Lisbon, Portugal (V. Borges)

**Keywords:** respiratory syncytial virus, respiratory infections, classification, genotype, phylogeny, epidemiology, genome, glycoprotein, RSV, HRSV, G, F, lineage, surveillance, orthopneumovirus, evolution

## Abstract

A globally implemented unified phylogenetic classification for human respiratory syncytial virus (HRSV) below the subgroup level remains elusive. We formulated global consensus of HRSV classification on the basis of the challenges and limitations of our previous proposals and the future of genomic surveillance. From a high-quality curated dataset of 1,480 HRSV-A and 1,385 HRSV-B genomes submitted to GenBank and GISAID (https://www.gisaid.org) public sequence databases through March 2023, we categorized HRSV-A/B sequences into lineages based on phylogenetic clades and amino acid markers. We defined 24 lineages within HRSV-A and 16 within HRSV-B and provided guidelines for defining prospective lineages. Our classification demonstrated robustness in its applicability to both complete and partial genomes. We envision that this unified HRSV classification proposal will strengthen HRSV molecular epidemiology on a global scale.

Human respiratory syncytial virus (HRSV) is a leading cause of acute lower respiratory tract infection in children, elderly, and immunocompromised persons. In 2023, the US Food and Drug Administration and the European Medicines Agency approved the first HRSV vaccines (*1*,*2*). Simultaneously, a monoclonal antibody was approved for widespread use in infants and not limited to high-risk and premature children (*3*). The availability of HRSV immunization highlights the role of molecular epidemiology as a tool to monitor their efficacy. Standards for HRSV nomenclature for sharing of viral isolates and sequences in databases have been published (*4*). Nevertheless, a standardized HRSV phylogenetic classification system has yet to be defined and implemented.

In 2022, HRSV was designated as *Orthopneumovirus hominis* species within the Pneumoviridae family. Below species level are 2 antigenic groups, known as HRSV subgroup A (HRSV-A) and B (HRSV-B), that were previously referred to as subtypes (*4*–*6*). Within each subgroup, genotypes were initially defined based on statistically supported phylogenetic clades inferred with the second hypervariable region of the G gene (Figure 1, panels A, B) (*7*). The G gene, encoding the attachment glycoprotein, exhibits the highest genetic and antigenic variability. Of note, the gene has undergone a duplication of a 72-nt fragment in HRSV-A and 60-nt fragment in HRSV-B (Figure 1, panel B) (*8*,*9*).

**Figure 1:**
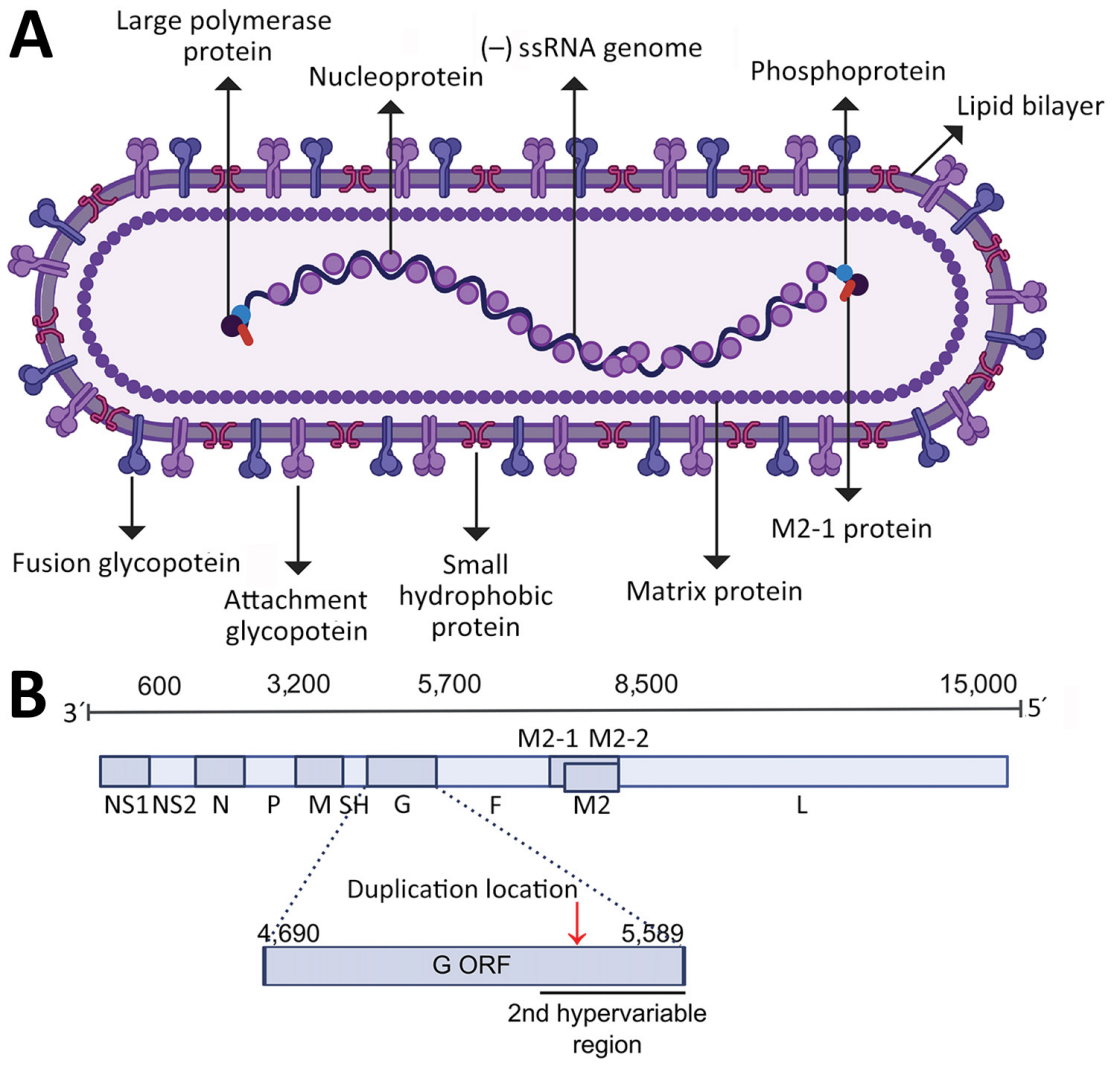
The structure and genome of human respiratory syncytial virus (HRSV). A) Schematic of the HRSV virion structure detailing the location of structural proteins. B) Schematic of the HRSV genome organization with the approximated location of genes highlighted; the exact location slightly differs between subgroups and strains. The location of the second hypervariable region in the G gene, used originally for molecular epidemiology classification, is detailed. Red arrow in panel B indicates location of the G gene 72-nt duplication in HRSV-A and 60-nt duplication in HRSV-B. Figure created with BioRender (https://www.biorender.com). F, fusion glycoprotein; G, attachment glycoprotein; L, large polymerase protein; M, matrix protein; M2, M2 protein; N, nucleocapsid; NS, nonstructural protein; ORF, open reading frame; P, phosphoprotein; SH, small hydrophobic protein.

To identify emerging genotypes, researchers have used genetic distances between phylogenetic clades and distinctive genetic features, accompanied by variable nomenclature based on the gene (GA1–GA7 in HRSV-A and GB1–GB4 in HRSV-B), country and subgroup (SAB1–SAB4 for South African genotypes in HRSV-B), or city and province (NA1–NA2 [Niigata] and ON1 [Ontario] in HRSV-A, BA1–BA9 [Buenos Aires] in HRSV-B) (*7*–*16*). Since 2020, alternative phylogenetic reclassifications have been proposed; Goya et al. established a hierarchical classification system for HRSV phylogenies, comprising genotypes, subgenotypes, and lineages, using the G gene (*17*). That framework enabled laboratories without capacity for whole-genome sequencing to conduct molecular epidemiology studies. Independently, Ramaekers et al. (*18*) proposed reclassifications into lineages and Chen et al. (*19*) into genotypes using complete HRSV genomes. Those approaches support comprehensive monitoring of viral evolution across all genes, including the F gene encoding the fusion protein, a crucial target for monoclonal antibodies and the foundation of approved HRSV vaccines (Figure 1, panel A). Of note, challenges in HRSV molecular epidemiology persisted within the reclassification-defined categories because of reliance on genetic or patristic distances between tree tips or nodes.

The milestones achieved in HRSV interventions have renewed interest in addressing the challenge of classifying HRSV below the subgroup level. Those advances prompted establishment of the HRSV Genotyping Consensus Consortium (RGCC), formed by HRSV and virus evolution experts aiming to provide standardized criteria for harmonizing global HRSV molecular surveillance efforts. We present a novel framework for HRSV classification below the subgroup level, based on current knowledge of HRSV diversity and evolution, focused on practical implementation for molecular epidemiology.

## Methods

### HRSV Sequences Dataset

We downloaded HRSV complete genomes from the National Center for Biotechnology Information Virus (https://www.ncbi.nlm.nih.gov/labs/virus) and GISAID EpiRSV (https://www.gisaid.org) databases through March 11, 2023, using a filter for sequence length >14,000nt, obtained from human hosts and including the year and country of the sample collection (Appendix 1 Figure 1). We reserved sequences containing nucleotide ambiguities, indicating inadequate sequencing depth, for epidemiologic analysis but excluded them from formal lineage definition (Appendix 1).

We aligned sequences with MAFFT version 7.490, and inspected and corrected alignment artifacts with Aliview version 1.28 (https://ormbunkar.se/aliview), mainly in the G gene (*20*,*21*). We trimmed alignment ends to encompass complete genomes from the first codon of the first gene (NS1) to the last codon of the last gene (L). We considered partial genomes if the lack of sequence was within 50 nt of the genome ends. We used RSVsurver (https://rsvsurver.bii.a-star.edu.sg) to identify and remove genomes with nucleotide insertions or deletions causing frameshift in any open reading frame. After alignment trimming, detection of identical sequences prompted redundancy removal using BBmap (https://jgi.doe.gov/data-and-tools/software-tools/bbtools), resulting in the final set of 1,538 HRSV-A and 1,387 HRSV-B genomes (Appendix 1 Figure 1).

### Phylogenetic Analysis

We constructed maximum-likelihood phylogenetic trees with IQ-TREE version 2.2.0 (http://www.iqtree.org) (Appendix 1). We considered monophyletic clades statistically supported when SH-aLRT value was >80% and UFBoot2 value was >90% (*22*,*23*) (Appendix 1). We assessed temporal signal with TempEst version 1.5.3 (http://tree.bio.ed.ac.uk/software/tempest), and we inferred molecular-clock phylogenies with TreeTime (https://github.com/neherlab/treetime) (*24*).

We inferred the ancestral sequence reconstruction using Augur bioinformatic toolkit version 23.1.0 (https://docs.nextstrain.org/projects/augur/en/23.1.0) (*25*). We assessed recombination events by alignment-based method using RDP4 (http://web.cbio.uct.ac.za/~darren/rdp.html) and phylogenetic-based TreeKnit (https://pierrebarrat.github.io/TreeKnit.jl) (Appendix 1). We inferred the amino acid substitutions linked to the clades in the tree using Augur and automated the initial screening of lineages with Autolin (*26*). We manually curated amino acid comparison among monophyletic clusters to rectify conflicts arising from internal (nested) lineages and the confirmation of the lineage-defining amino acids in >90% of the clade’s sequences. Results are available at https://github.com/rsv-lineages/Classification_proposal.

## Results 

### Baseline Agreements on the HRSV Classification Definition

Our proposed classification establishes HRSV lineages for viruses below subgroup level. Studies have shown that HRSV phylogenetic trees constructed with complete genomes exhibit superior resolution (*17*–*19*). Therefore, we defined a classification system based on maximum-likelihood phylogenetic trees inferred from complete HRSV genomes. The maximum-likelihood algorithm formulates hypotheses about the evolutionary relationships among sequences; the implementation within IQ-TREE dealing with large datasets makes it particularly well suited to assert HRSV genomic phylogeny including sequences collected >50 years ago (*22*). We defined complete HRSV genomes to the nucleotide sequences spanning from the first codon of the first gene (NS1) to the last codon of the last gene (L). We considered almost-complete genomes if the sequence information gaps were within a 50-nt window at the genome ends. To define lineages, we only used genomes without nucleotide ambiguities (in accordance with the IUPAC code for nucleotide degeneracy).

### Genomic Dataset Used for Lineages Definition

**Figure 2:**
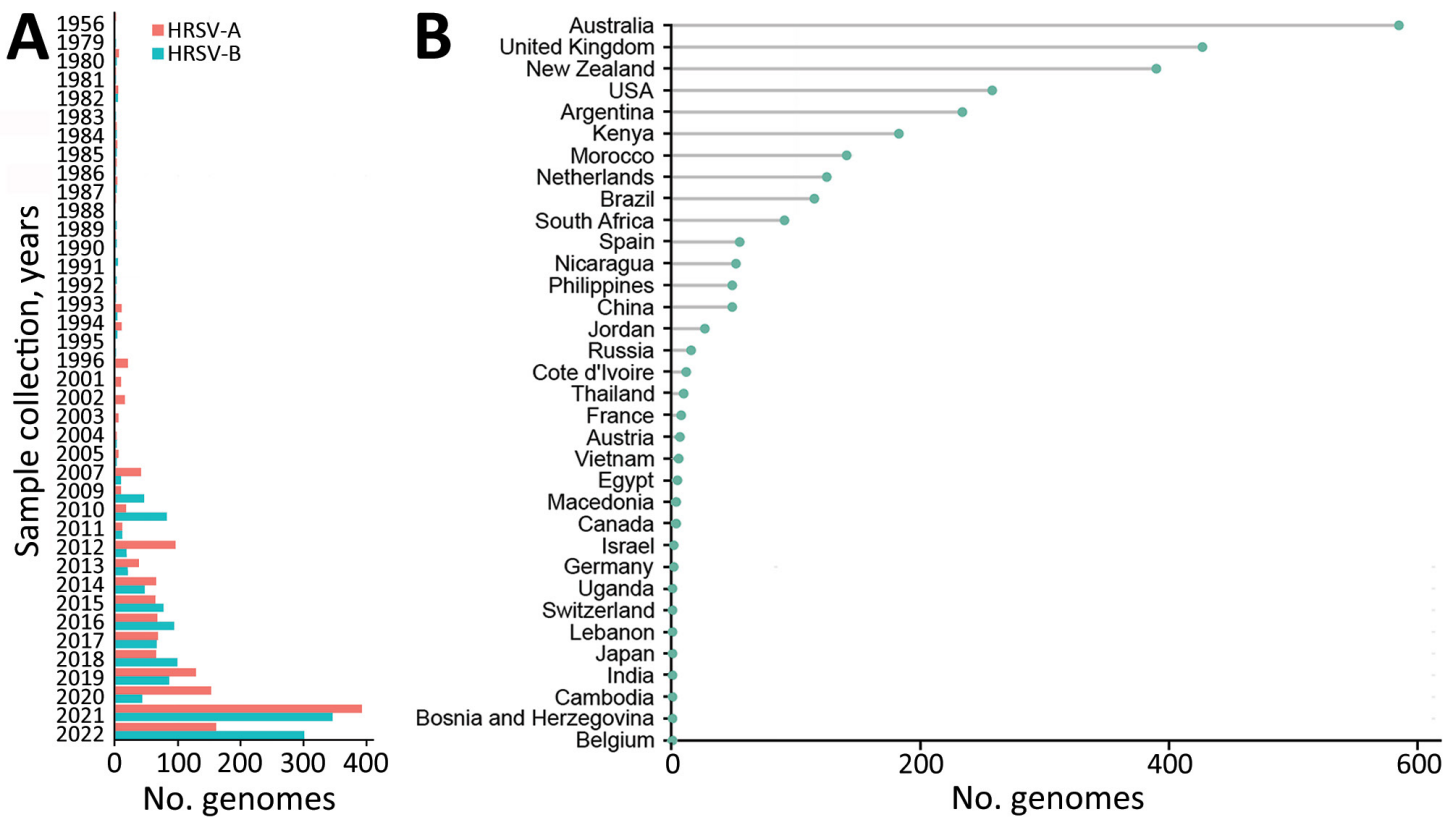
The global HRSV genomics surveillance landscape. HRSV genomes from GenBank and GISAID (https://www.gisaid.org) databases through March 11, 2023, that met inclusion criteria used for classification are shown by year of sample collection and subgroup (A) and by country of origin (B). HRSV, human respiratory syncytial virus.

Applying the established baseline agreements, we gathered 1,538 HRSV-A and 1,387 HRSV-B high-quality genomes from public databases. The dataset revealed a limited global HRSV genomic surveillance; <20 genomes deposited annually through 2007 (Figure 2, panel A; Appendix 1 Figure 2). Since 2008, the number of genomes and representation of countries improved; a surge occurred after 2021, probably driven by expansion of viral genomics since the SARS-CoV-2 pandemic and the approval of the HRSV prophylactic treatments (Figure 2, panel A; Appendix 1 Figure 2). Considering delays in genome deposition in public databases, the number of genomes in 2022 may be higher than those used in this study. Regarding geographic representation, 9 countries (Australia, United Kingdom, New Zealand, United States, Argentina, Kenya, Morocco, Netherlands, and Brazil) submitted >100 genomes; only the United Kingdom achieved uninterrupted surveillance since 2008, but Australia deposited the most genomes globally (Figure 2, panel B).

### Accurate Root Placement in HRSV Phylogenetic Trees

**Figure 3:**
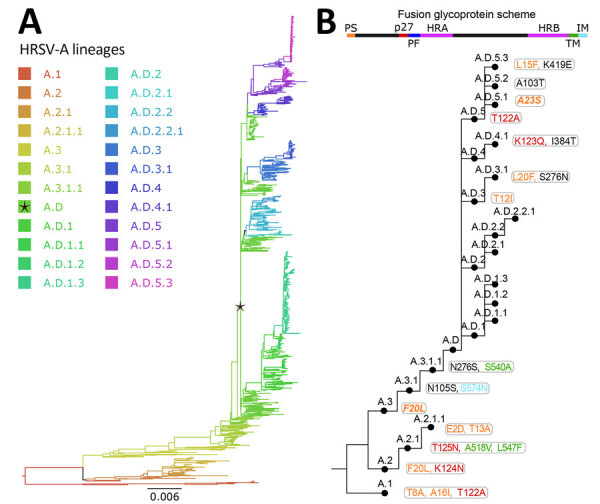
Human respiratory syncytial virus A lineage classification. A) HRSV-A maximum-likelihood phylogenetic tree (1,480 sequences), colored by lineage classification. Black star indicates A.D lineage, defined by the 72-nt duplication in the G gene. Scale bar indicates substitutions per site. B) Simplified scheme of the lineage designation to highlight the presence of nested lineages. The amino acid changes in the F glycoprotein are listed next to lineage name and colored according to their location in the fusion protein.

We reconstructed maximum-likelihood phylogenetic trees for the HRSV-A and HRSV-B datasets. We used 2 approaches to root the trees: the use of an outgroup, a conventional method for inferring the tree root using sequences known to be evolutionarily distant; and phylodynamic analysis, integrating temporal and phylogenetic patterns in virus evolution (Appendix 1). Both approaches consistently identified the same root for each subgroup cluster (Appendix 1 Figure 3). Phylodynamic analysis also identified 58 outlier sequences for HRSV-A and 2 for HRSV-B that were excluded from lineage designation. The final dataset considered for lineage designation comprised 1,480 HRSV-A and 1,385 HRSV-B genomes (Appendix 2 Table).

### HRSV Lineage Definition

We defined HRSV lineage as a statistically supported monophyletic cluster comprising >10 sequences and characterized by >5 aa substitutions, compared to the parental lineage. The lineage-defining amino acids, present in >90% of the sequences within the clade, may be found in any of the viral proteins.

Phylogenetic classifications vary among viral species aiming to define clusters reflecting the heterogeneity of the viral population, considering each virus unique evolutionary characteristics and using arbitrary thresholds for long-term applicability (*27*–*29*). Inherent bias exists in any classification system because of availability and spatiotemporal representation sequences. Therefore, our HRSV lineage definition did not include criteria of sequences from different outbreaks or countries to enable early detection of novel lineages. However, we propose establishing a threshold of >10 genomes for defining a lineage to monitor HRSV strains circulating within communities.

We observed the presence of distinctive signature amino acids shared by sequences of a phylogenetic clade in comparison to the parental lineage is a simple method to identify a new lineage. Methods (i.e., average nucleotide genetic distances, average patristic distances, or patristic distances between nodes) need phylogenies with complete datasets to define new categories, becoming complex with rapid increases of available sequences (*16*–*19*). In our proposal, we initially screened different amino acid thresholds in an automated manner, ranging from 1–10 lineage-defining amino acids (Appendix 1). The number of small lineages decreased as the number of lineage-defining amino acids increased, and 5 amino acids resulted in an intermediate complexity of lineages defined for both HRSV subgroups. Furthermore, we proposed that the lineage-defining amino acids should be conserved in >90% of the genomes within a clade, considering the potential reversion in some of the genomes within highly mutated hotspot sites. We acknowledged that other numbers of genomes or amino acids thresholds could be useful, but we emphasized that the key to establishing a global consensus is clear operational guidelines and a robust classification, 2 aspects that our proposal fulfills.

### HRSV Lineage Nomenclature

**Figure 4:**
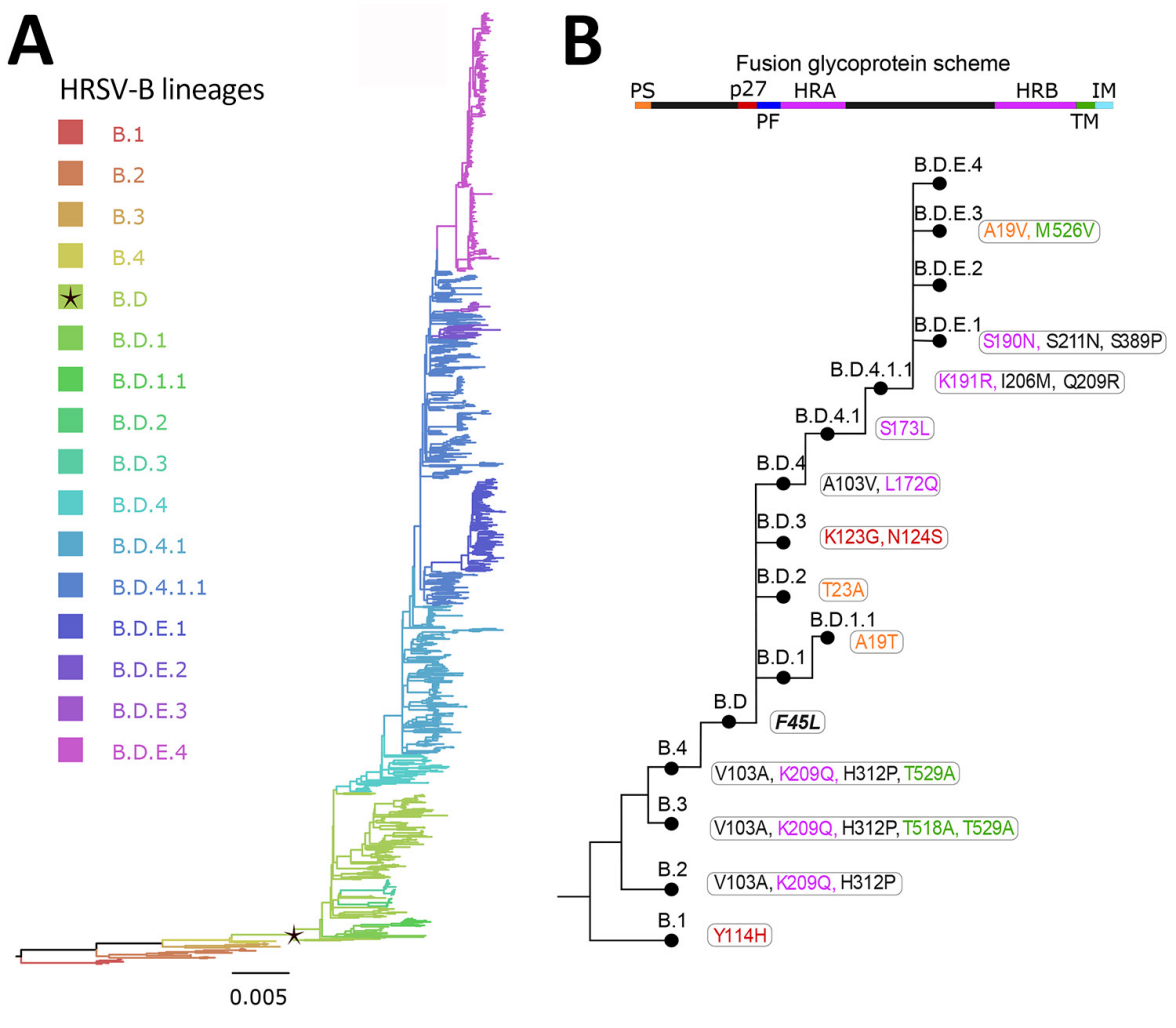
Human respiratory syncytial virus B lineages classification. A) HRSV-B maximum-likelihood phylogenetic tree (1,385 sequences), colored according to lineage classification. Black star indicates B.D lineage, defined by the 60-nt duplication in the G gene. Scale bar indicates substitutions per site. B) Simplified scheme of the lineage designation to highlight the presence of nested lineages. The amino acid changes in the F glycoprotein are listed next to lineage name and colored according to their location in the fusion protein.

We defined the lineage nomenclature integrating the HRSV subgroup letter and ascending ordinal numbers, separated by dots to represent nested lineages (Figure 3, panels A, B; Figure 4, panels A, B). Furthermore, we assigned a distinct nomenclature to the 72-nt (24-aa) G-gene duplication within HRSV-A and 60-nt (20-aa) G-gene duplication within HRSV-B. Those genetic events are epidemiologically relevant, because only viruses with G-gene duplication have been detected since 2017 (*30*–*33*). To track those viruses, we used the alias D, specifically A.D (historically, ON1 genotype) for HRSV-A and B.D (historically, BA genotype), for HRSV-B and nested lineages with increasing ordinal numbers. In summary, letters A and B indicate the HRSV subgroup at the beginning of the lineage name, C is unused, and D serves as an alias for 72-nt and 60-nt duplication within the G gene. In addition, aliases starting from E are limited to 3 numerical levels of nested lineages, preventing indefinite accumulation of numbers. For example, B.D.4.1.1 lineage has descendant lineages named B.D.E.1–B.D.E.4 instead of B.D.4.1.1.1–B.D.4.1.1.4, where E represents 4.1.1 (Figure 4, panels A, B). The nomenclature is based on the tree topology, reflecting the order of the nodes from the root to the tips, but it is unrelated to the sequence collection date or date of the most recent common ancestor of the lineage.

To remain functional, a nomenclature system requires periodic updates as new lineages emerge. Therefore, we have established 2 open repositories on GitHub containing definitions of each lineage, signature mutations, and representative sequences. The repositories are available at https://github.com/rsv-lineages/lineage-designation-A and https://github.com/rsv-lineages/lineage-designation-B; they are intended to provide up-to-date definitions and serve as a platform for discussion and designation of novel lineages.

### Lineages within the HRSV-A and HRSV-B Rooted Trees

We reconstructed ancestral sequences at the root of the phylogenetic trees. Although the sequences are not biologically real, they served as surrogate parental lineages during initial classification. Identifying monophyletic clusters with >10 sequences and >5 aa changes compared with the reconstructed root sequence, we defined 3 HRSV-A lineages (A.1–A.3) and 4 HRSV-B lineages (B.1–B.4). We were unable to classify 2 sequences, EPI-ISL-15771600_USA_1956 (GISAID) and MG642074_USA_1980 (GenBank), perhaps because they belong to underrepresented extinct lineages.

We further analyzed the first lineages in an iterative manner to identify nested lineages; as a result, we identified a total of 24 lineages within HRSV-A, and 16 within HRSV-B (Figure 3; Figure 4). Close to the root of the HRSV-B tree, extinct lineages were underrepresented, comprising <10 sequences but featuring >5 distinct amino acids (B.1, B.3, B.4). Despite the low number of sequences, we included them as lineages to trace evolutionary branches that gave rise to currently circulating lineages. In addition, A.D.2 is slightly below the sequence threshold; nonetheless, we kept the lineage category to emphasize the common ancestor among A.D.2.1 and A.D.2.2.

We scrutinized the presence and absence of the duplication in the G gene across each tree. Although patterns were mostly as expected with a single historical duplication event, some genomes within the clade with the duplication in G lacked the duplication. The dispersed association of these sequences in the phylogenetic tree, rather than the monophyletic cluster we expected, suggests the virus did not lose the nucleotide duplication (Appendix 1 Figure 4). Instead, similar read length to the duplication region of certain short-read next-generation sequencing technologies potentially masked the presence of the duplication when used in the consensus genome assembly with reference sequences that do not possess the nucleotide duplication. Therefore, we recommend using such data with quality filtered reads of a length >150nt to avoid this problem.

Lineage-defining amino acids were present in all HRSV proteins, primarily identified within the G protein (Tables 1, 2). Also, the lineage-defining amino acids at polymerase L protein were noteworthy, contributing to the distinction of 21 of 24 HRSV-A lineages and 15 of 16 HRSV-B lineages (Tables 1, 2). Of interest, the F protein contributed to define 14 lineages in HRSV-A and 13 in HRSV-B (Figure 3, panel B; Figure 4, panel B). The G and F surface glycoproteins are likely under selection pressure from antibody-mediated immunity and exhibit a robust phylogenetic signal (*18*,*31*). Whereas the G protein displays substantial nucleotide and amino acid sequence plasticity, the F protein experiences strong negative selection, likely attributed to functional or structural constraints (*34*). For instance, the fusion peptide is the only region in F without lineage-defining amino acids (Figure 3, panel B; Figure 4, panel B). Although the low diversity of the F protein is promising for HRSV interventions, monitoring the F protein during global implementation is essential to estimate the antigenic impact of amino acid substitutions.

**Table 1:** Amino acids defining lineages in human respiratory syncytial virus A*

Lineage	NS1	NS2	N	P	M	SH	G	F	M2–1	M2–2	L
A.1	105(I)L	38(R)K	216(Y)H	73(V)A†	91(C)S		15(K)R; 38(I)V; 71(L)S; 74(A)T; 101(F)P; 104(L)P; 111(T)I; 121(R)G; 141(I)A; 157(N)S; 215(L)P; 222(P)S; 233(K)E; 250(N)S; 254(G)R; 258(H)L; 286(K)M; 304(Y)S; 310(L)P†; 317(S)P; 321(N)R	8(T)A; 16(A)I; 122(T)A		18(N)T; 44(Q)P; 51(P)L; 80(D)E	81(I)L; 104(T)A; 162(L)S; 232(I)T; 240(N)S; 255(H)N; 445(V)I; 603(N)D; 754(R)G; 821(V)I; 1180(F)L; 1657(Y)H; 1718(T)A; 1731(D)E; 1756(T)A; 1773(T)M; 1778(K)R; 1969(T)M; 2019(V)A
A.2							4(N)T; 107(I)T/N; 121(R)S; 123(K)E; 225(V)A; 244(I)R; 251(N)S; 256(P)L†; 263(F)L; 286(K)E†; 314(S)P†	20(F)L; 124(K)N	120(L)P; 179(L)S	1(M)T†; 25(R)S†; 54(T)P; 68(T)A	174(D)G; 237(Q)H; 1551(K)R; 1721(I)V; 1723(S)C
A.2.1		7(D)G	64(L)V	73(V)A			57(A)V; 108(T)I†; 161(N)D; 303(V)I; 321(N)D	125(T)N; 518(A)V; 547(L)F	182(S)N	48(I)T	148(D)N†; 1724(N)D
A.2.1.1							117(L)P†; 124(S)P; 146(P)L; 206(P)Q	2(E)D; 13(T)A			104(T)A†; 144(G)E; 257(E)D†; 1757(M)T; 1847(N)S
A.3			84(R)K				4(N)T; 15(K)R; 107(I)T; 111(T)P†; 289(F)L	20(F)L†	120(L)P; 179(L)S	1(M)T; 54(T)P; 68(T)A; 69(I)T	224(N)S; 237(Q)H/Y; 1551(K)R; 1721(I)V; 1764(K)R
A.3.1		38(R)K					106(E)G†; 111(P)S; 237(N)D; 286(K)E; 316(P)S	105(N)S; 574(S)N	125(I)V†	25(S)N; 39(F)I; 50(S)P; 52(N)D	6(N)S†; 177(H)Q†; 216(N)S; 388(V)I; 575(E)D; 754(R)K; 1180(F)L; 1471(H)N; 1700(N)S†; 1730(N)S; 1745(L)S; 2135(Y)H; 2163(H)N
A.3.1.1							142(Q)L; 208(I)L; 297(N)Y	276(N)S; 540(S)A			103(I)T; 240(N)S; 1725(D)E/G; 2163(N)Y
A.D							232(E)G†; 253(T)K†; 260dup>QEETLHSTTSEGYLSPSQVYTTSG				598(Y)H†
A.D.1					43(M)I		142(L)S; 320 (T)A†				143(N)D; 179(T)S/L; 1653(I)V; 1661(K)N
A.D.1.1							121(S)G; 273(Y)H			2(T)I	124(V)I; 234(I)V; 970(N)S; 1187(D)G; 1236(T)S
A.D.1.2			104(I)F		25(K)R†	51(H)Q	129(T)I†; 174(S)N				1789(S)F
A.D.1.3	9(I)L	3(T)I					100(S)I†; 225(V)A; 263(E)Q; 265(L)P; 270(S)P†; 277(S)P; 280(Y)H; 291(S)P; 297(Y)H; 310(L)P; 316(S)P†; 321(K)X; 753(D)G†				755(D)G†; 2111(N)H
A.D.2							134(K)I; 262(E)K			46(N)S	598(H)Y†; 1731(D)G
A.D.2.1				69(T)A		21(I)V	67(H)N; 107(T)P; 120(P)T; 240(K)R; 280(Y)H; 287(E)D; 294(S)P; 303(V)I; 319(T)S				144(G)E; 610(P)S; 1599(H)R
A.D.2.2							240(R)K; 243(I)S; 303(I)V				1731(D)G; 1950(I)L
A.D.2.2.1							224(E)G; 265(L)I; 274(L)P				171(L)P; 256(K)R
A.D.3				92(T)M†			113(T)I; 131(V)D†; 178(N)G†; 258(H)Q†; 266(H)L†	12(T)I		79(T)A	835(L)M
A.D.3.1							101(F)S; 151(R)H; 225(V)A; 230(P)T†; 250(S)F†; 263(E)K	20(L)F; 276(S)N		48(I)L	
A.D.4				63(I)V/A	47(L)Q		57(A)T†; 206(P)Q; 310(L)P†				1726(K)R
A.D.4.1			122(E)D				90(Y)H; 111(S)F†; 283(S)P; 312(Q)X; 319(T)A	123(K)Q; 384(I)T	9(F)Y; 173(I)V	1(T)M	1617(V)I
A.D.5							206(P)Q; 303(V)A†; 320(T)A†	122(T)A			1723(S)G
A.D.5.1			216(Y)H				57(A)V; 118(T)I†; 209(K)R; 319(T)I	23(A)S†			
A.D.5.2				69(T)I			57(A)V; 209(K)R†	103(A)T†		26(C)Y†	
A.D.5.3		3(T)A; 8(T)A					189(I)T; 225(V)A; 243(I)T; 276(P)Q†; 280(Y)H; 284(G)D†; 314(L)P†	15(L)F; 419(K)E	52(K)R; 173(I)V†	27(R)K; 64(D)E	114(R)K; 179(T)A; 335(V)I; 1051(N)S

*The lineage-defining amino acids within each viral protein are detailed. This description provides the location of the amino acid using the genome coordinates hRSV/A/England/397/2017 (lineage A.D.2.2.1, National Center for Biotechnology Information Virus accession no. PP109421.1, GISAID EpiRSV accession no. EPI_ISL_412866), followed by the amino acid found in the parental lineage in parentheses. Finally, the amino acid substitution that drives it to become a lineage is specified. X represents an amino acid deletion. NS, nonstructural protein; N, nucleocapsid; P, phosphoprotein; M, matrix protein; SH, small hydrophobic protein; G, attachment glycoprotein; F, fusion glycoprotein; M2, M2 protein; L, large polymerase protein †Amino acid changes detected in not all but >90% of the lineage sequences.

**Table 2:** Amino acids defining lineages in human respiratory syncytial virus B*

Lineage	NS1	NS2	N	P	M	SH	G	F	M2–1	M2–2	L
B.1							103(K)I; 133(T)P; 222(K)D	114(Y)H			148(E)D; 948(N)H; 1177(I)M; 1613(K)N; 2014(A)S
B.2	124(D)N	24(E)D		66(I)T			95(S)P†; 118(I)T; 137(I)T; 150(S)P; 206(I)T; 220(M)T; 221(P)T; 275(S)L†; 283(Y)S; 289(S)P; 298(I)T; 311(X)Q†; 314(X)Q	103(V)A†; 209(K)Q; 312(H)P		41(Y)H; 49(C)R†; 68(T)A	308(I)V; 1742(T)M; 2021(N)D
B.3							4(H)N; 77(Q)S; 101(S)P; 137(I)T; 140(S)P; 150(S)P; 152(S)P; 154(N)I; 206(I)T; 221(P)L; 283(Y)S; 298(I)T; 311(X)Q	103(V)A†; 209(K)Q; 312(H)P; 518(T)A†; 529(T)A			184(T)N; 308(I)V; 1043(I)V; 1547(K)R; 1700(N)D; 1764(K)R; 2021(N)D; 2042(T)I
B.4	45(A)T; 105(M)I†				89(I)T	57(L)Q	4(H)N; 32(R)K; 101(S)P; 109(S)P; 137(I)T; 140(S)P; 143(T)N; 150(S)P; 152(S)P; 205(T)P; 206(I)T; 217(P)L; 221(P)L; 235(L)P; 283(Y)S; 298(I)T; 311(X)Q	103(V)A; 209(K)Q; 312(H)P; 529(T)A			184(T)N; 308(I)V; 1043(I)V; 1250(G)S; 1700(N)D; 1726(S)R; 1764(K)R; 2021(N)D; 2042(T)I; 2065(K)N
B.D	138(N)H		372(A)T†				138(T)S†; 157delKK†; 221(L)P†; 227(T)I†; 239dupERDTSTSQSTVLDTTTSKHT; 285(H)Y†; 311(X)Q†; 314(X)Q†	45(F)L†	142(N)S†		177(H)Y; 1716(M)I†; 1787(A)E†
B.D.1		82(T)S			89(T)I		290(E)G†; 311(Q)X†				1593(N)S
B.D.1.1				63(I)V			75(T)S; 204(P)T; 217(L)P; 227(I)T	19(A)T			59(M)I; 1250(G)S
B.D.2				145(I)V			87(I)T; 178(Q)R†; 239(E)K	23(T)A	181(V)I		1250(G)S; 1588(I)L
B.D.3		4(T)K; 80(K)T†					87(I)T; 217(L)P; 268(I)T; 285(Y)H; 299(P)S; 311(Q)X	123(K)G; 124(N)S	188(T)P	3(K)N; 43(H)Y	1489(A)V; 1588(I)L
B.D.4						49(T)I	198(I)T	103(A)V; 172(L)Q	181(V)I		
B.D.4.1			216(H)Y†				107(T)A; 136(R)T; 252(T)I†; 279(I)T	173(S)L			715(I)V†; 1712(T)A
B.D.4.1.1						64(N)D†	131(A)T†; 137(T)I†; 288(T)I†	191(K)R; 206(I)M; 209(Q)R			1479(V)A†; 1716(I)V†
B.D.E.1							100(S)G†; 214(P)S; 221(P)L; 252(I)T; 256(K)N†; 268(I)T; 275(S)P; 285(Y)H	190(S)N; 211(S)N; 389(S)P		27(M)T†; 35(D)N; 49(C)F	570(K)R†; 1759(R)K
B.D.E.2			303(I)V				176(N)S; 269(A)V; 274(T)A†				1707(H)N
B.D.E.3		1(M)V					104(Q)H; 128(H)Y; 135(G)S; 141(T)K; 299(P)S†; 311(X)Q†	19(A)V; 526(M)V		75(H)Y†	1965(V)A†
B.D.E.4				61(N)T			135(G)S†; 138(S)F†; 300(T)A; 303(E)G				164(V)M; 309(V)L; 824(I)M†; 1489(A)T/M; 1732(C)Y

*The lineage-defining amino acids within each viral protein are detailed. This description provides the location of the amino acid using the genome coordinates HRSV/B/AUS/VIC-RCH056/2019 (lineage B.D.4.1.1, National Center for Biotechnology Information Virus (https://www.ncbi.nlm.nih.gov/labs/virus) accession no. OP975389.1, GISAID EpiRSV (https://www.gisaid.org) accession no. EPI_ISL_1653999), followed by the amino acid found in the parental lineage in parentheses. Finally, the amino acid substitution that drives it to become a lineage is specified. X represents an amino acid deletion NS, nonstructural protein; N, nucleocapsid; P, phosphoprotein; M, matrix protein; SH, small hydrophobic protein; G, attachment glycoprotein; F, fusion glycoprotein; M2, M2 protein; L, large polymerase protein. †Amino acid changes detected in not all but >90% of the lineage sequences.

### Using G and F Sequences with the HRSV Lineage Classification System

**Figure 5:**
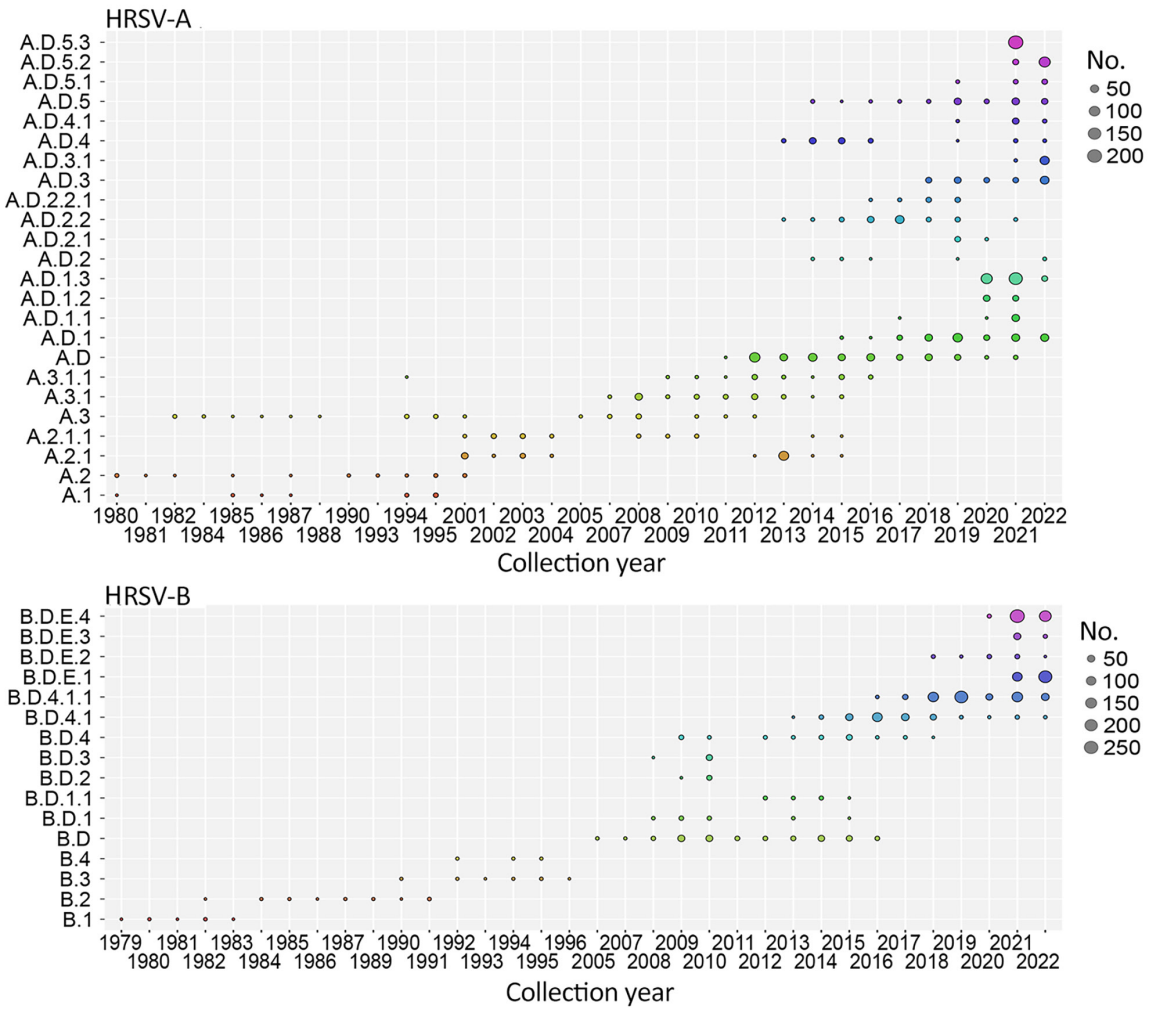
Temporal distribution of HRSV-A and HRSV-B lineages. A total of 2,744 HRSV-A genomes and 2,443 HRSV-B genomes available in public databases through March 2023 were included. HRSV, human respiratory syncytial virus.

The main challenge for global expansion of HRSV genomics is the absence of a cost-effective, globally standardized and validated methodology for sequencing, in contrast to SARS-CoV-2 or influenza virus (*35**,**36*). In addition, limited funding and infrastructure cause some laboratories to prefer sequencing the G gene only (*37*–*39*). Although we highly recommend using complete genomes for HRSV lineage assignment to ensure the maximum accuracy of the classification and monitor the amino acid changes in all viral proteins, partial genomes covering the G and F genes can be used because overall they reproduce the topology of the HRSV tree (*17*,*18*). We do not recommend the use of smaller G gene regions such as the second hypervariable region (250-nt length at the 3′ gene end) (Figure 1) that was used historically for molecular epidemiology because previous reports showed a decreased phylogenetic signal (*17*). The use of G, F, or both genes for lineage classification should rely on phylogenetic associations with reference sequences. Of note, using only G and F genes is inadequate for defining novel lineages because of the inability to detect lineage-defining amino acids across all viral proteins. Our analysis showed minimal misclassification (1.2%) in HRSV-A and none in HRSV-B when using only the G gene (Appendix 1 Figure 5). However, the G ectodomain alone resulted in an 18.86% misclassification rate for HRSV-A and none for HRSV-B. The F gene alone had misclassification rates of 38.18% for HRSV-A and 1.23% for HRSV-B because of polytomies affecting lineage assignments within A.D.1 and A.D.5. Combining G and F gene fragments reduced misclassification to 0.07% for HRSV-A and none for HRSV-B, indicating that this approach provides optimal resolution for both subgroups (Appendix 1 Figure 5).

### Prospective HRSV Lineage Assignment and Definition

Assigning sequences to the existing lineages can be automated using online tools such as NextClade (https://clades.nextstrain.org) (*40*), ReSVidex (https://cacciabue.shinyapps.io/resvidex_wg), INSaFLU (https://insaflu.insa.pt) (*41*), or UShER (https://usher.bio) (*42*). However, to define a novel lineage, we encourage users to follow our guidelines (Appendix 1), available on GitHub (https://github.com/orgs/rsv-lineages/repositories). We anticipate new lineages of HRSV-A/B will continue to emerge, and we envision updating our proposed nomenclature to incorporate new lineages. We encourage reporting of new HRSV lineages at the RGCC GitHub page as an issue within the corresponding repository for HRSV-A/B. The RGCC study group will evaluate the newly proposed lineage and update reference alignments if confirmed.

 Importantly, assigning the lineage of a query sequence does not require the use of complete genomes or the absence of nucleotide ambiguities; rather, it requires a supported association within a phylogenetic clade. However, defining a new lineage requires the use of complete genomes without ambiguities, because amino acid characterization of all viral proteins is essential.

### Molecular Epidemiology of HRSV with Proposed Classification

We described the HRSV molecular epidemiology including all available genomes, even those previously discarded during the dataset curation. We analyzed the seasonality of lineages using a dataset comprising 2,277 HRSV-A and 2,058 HRSV-B genomes, revealing notable co-circulation and lineage replacement over time (Figure 5). In HRSV-A, A.1 and A.2 lineages are extinct: the last detected sequences of A.1 were collected in 1995 and of A.2 in 2015. Since 2011, A.D and nested lineages continue to circulate; A.D.2.2 and A.D.4 were detected in 2013, indicating rapid divergence of the HRSV-A viruses with the 72-nt duplication in G gene. In HRSV-B, lineages B.1, B.2, B.3, and B.4 exhibited strong lineage replacement (Figure 5). Although the B.D lineage with a 60-nt duplication in the G gene (B.D lineage) was detected in 1999, complete genomes became available in 2005 (*8*). By 2009, only B.D and nested lineages were detected, and since 2017, only B.D.4 and nested lineages have been observed.

HRSV lineages may have been underrepresented before the COVID-19 pandemic because of limited genomic surveillance. However, our classification system allows for updates if prepandemic genomes meeting lineage criteria are shared. Some lineages, such as A.D.3.1, A.D.5.2, and A.D.5.3 in HRSV-A and B.D.E.1 and B.D.E.3 in HRSV-B, appear to be exclusive to the postpandemic period, although most of their lineage-defining amino acid were present in parental prepandemic strains. For instance, A.D.5.2 was recognized as a distinct lineage with the emergence of the C26Y substitution in M2–2, whereas other signature amino acids were present in a 2019 parental lineage genome (GenBank accession no. MZ515825). Detection of postpandemic lineages does not contradict studies reporting no new post-pandemic genotypes because those studies relied on earlier classification systems (*43*–*46*). The possibility that these new lineages circulated before the pandemic depends on the deposition of genomes.

Some of the lineages were detected in specific countries (Appendix 1 Figure 6). For example, A.D.1 descendant lineages, A.D.5.3 and most of B.D.E.4 cases were identified in Australia or New Zealand. Contemporary lineages such as B.D.4.1.1 and descendants B.D.E.1 and B.D.E.3, predominantly consisted of sequences from the United Kingdom. Global genomic surveillance bias presents a major confounding factor in lineage geodetection; for instance, most of the earliest lineages were detected in the United States, the principal contributor of HRSV genomes until 2007 (Appendix 1 Figures 2, 6).

## Discussion 

Consensus classification of HRSV below the subgroup level has been a challenge for multiple decades. Collaboratively, the HRSV molecular evolution research community, along with experts in the evolution of other respiratory viruses, have worked toward establishing a unified global classification system in the initiative HRSV Genotyping Consensus Consortium (RGCC). Our proposal categorizes HRSV-A/B sequences into lineages based on phylogenetic associations and amino acid markers, relying on complete genomes. Partial or low-quality genomes can be assigned to the existing lineages, emphasizing the robustness of this system. We developed standard guidelines for lineage definition and assignment and created online resources for updates, ensuring long-term utility. Defining a viral category below species through a phylogenetic-based classification is challenging; the system must exhibit reproducibility, balance complexity, and be updatable to capture the level of heterogeneity useful for viral surveillance. Our proposal addresses those requirements comprehensively.

HRSV is not an emerging virus; it generates annual outbreaks with co-circulation and replacement in the prevalence of its antigenic subgroups. Although some HRSV genomes were collected from clinical samples >50 years ago, the largest increase in the number of genomes has occurred since 2021. A limitation of our definition is the uncertainty of the antigenic effect of individual amino acid substitutions on lineages. Hence, whole-genome surveillance together with the study of lineage-phenotype association are essential, as observed in genetic and antigenic characterization in influenza to estimate the effectiveness of immunization (*47*). In 2023, recombinant F protein vaccines were approved; as their implementation progresses, we will learn how the vaccines affect viral evolution. We expect our unification proposal for the phylogenetic classification of HRSV to support spatiotemporal comparative lineage surveillance and detection of emerging lineages. In addition, we anticipate studies of association between lineages and the severity of HRSV disease, as well as associations of particular lineages with patients’ demographic characteristics.

Appendix 1 Additional information about classification of human respiratory syncytial virus below the subgroup level.

Appendix 2 Genomes used for classification of human respiratory syncytial virus lineages HRSV-A and HRSV-B.
