# Supplementary material for: Standardized Phylogenetic Classification of Human Respiratory Syncytial Virus below the Subgroup Level
Source: Emerg Infect Dis. 2024 Aug;30(8):1631–41. doi: 10.3201/eid3008.240209 (PMC11286072; doi:10.3201/eid3008.240209)
Supplement: Appendix 1 — Additional information about classification of human respiratory syncytial virus below the subgroup level. [file 24-0209-Techapp-s1.pdf]

Article DOI: http://doi.org/10.3201/eid3008.240209

*EID cannot ensure accessibility for supplementary materials supplied by authors. Readers who have difficulty accessing supplementary content should contact the authors for assistance.*

# Standardized Phylogenetic Classification of Human Respiratory Syncytial Virus Below the Subgroup Level

## Appendix 1

The supplementary description of each section appears in the order of mention within the manuscript.

## Methods

### HRSV Sequences Dataset

We downloaded HRSV complete genomes available from NCBI GenBank (https://www.ncbi.nlm.nih.gov/labs/virus) and GISAID EpiRSV (https://gisaid.org/) up to March 11, 2023 using a filter for sequence length above 14,000-nt, obtained from human hosts and including the year and country of the sample collection. We categorized sequences into files based on their reported subgroup resulting in 2,744 HRSV-A and 2,443 HRSV-B genomes (Appendix Figure 1). We reserved sequences containing nucleotide ambiguities, indicative of inadequate sequencing depth, for epidemiologic analysis but we excluded them from formal lineage definition. We removed genomes with any nucleotide ambiguities using BBmap (reformat, version Jan-2021). To ensure diversity without redundancy, BBmap (dedupe, version Feb-2020) was used to remove identical sequences, preserving one representative.

We aligned the sequences with MAFFT v7.490, and we inspected and corrected the alignments with Aliview v1.28, mainly in the G gene (17,21). Furthermore, we trimmed alignment ends to encompass complete genomes from the first codon of the first gene (NS1) to the last codon of the last gene (L). We considered partial genomes if the lack of sequence was within 50-nt of the genome ends. We used RSVsurver to detect and remove genomes with

nucleotide insertions or deletions causing frameshift in any open reading frame(s) (https://rsvsurver.bii.a-star.edu.sg). Following alignment trimming, the presence of identical sequences with nucleotide differences in the trimmed region prompted another round of redundancy removal using BBmap tool, resulting in the final set of 1,538 HRSV-A and 1,387 HRSV-B genomes (Appendix Figure 1).

#### Phylogenetic Analysis

We constructed maximum likelihood phylogenetic trees with IQ-TREE v2.2.0, using ModelFinder to find the best nucleotide substitution model (*1*,*2*). The reliability of sequence clusters was evaluated with SH-aLRT (1,000 replicates) and UFBoot2 (10,000 replicates) (*3*). We considered monophyletic clades defining lineages when SH-aLRT value was ≥80% and UFBoot2 value was ≥90%. Phylogenetic trees were visualized with Figtree v1.4.4 and Auspice (*4*,*5*). We assessed the temporal signal with TempEst v1.5.3, and we inferred molecular-clock phylogenies with TreeTime (*6*,*7*).

We assessed recombination events with both alignment-based and phylogenetic-based methods. RDP4 software was used to detect and characterize the recombination events within the sequence alignments using the RDP, GENECONV, Maximum Chi Square and 3SEQ methods with default settings (*8*). The TreeKnit software assessed recombination based on topological differences between trees by comparison of phylogenies inferred with the 5′ and 3′ ends of the alignments (4,500-nt each, excluding the G gene) (*9*). The resulting tanglegram, available at https://github.com/rsv-lineages/Classification_proposal, was visualized in Auspice. Recombination assessment, using RDP4 software (alignment-based) and phylogenetic tree topology-based analysis, found no evidence of recombination among HRSV sequences. Consequently, no sequences were removed due to genetic recombination.

## Results

#### Accurate Root Placement in HRSV Phylogenetic Trees

We used two approaches to define the correct phylogenetic tree root: a) the utilization of an outgroup, a conventional method for inferring the tree root using sequences known to be evolutionarily distant, and b) phylodynamic analysis, integrating temporal and phylogenetic patterns in virus evolution.

In the first approach, five HRSV-B genomes with the earliest collection dates were aligned with the HRSV-A dataset, and vice versa (Appendix 1 Figure 3 panels A, C). As anticipated, the five sequences from the alternative subgroup formed a distantly related clade, serving as the outgroup for rooting each tree. The second approach, without outgroups, involved reconstructing dated phylogenetic trees for each subgroup dataset, and the phylogenetic root automatically inferred by incorporating temporal information (Appendix Figure 3 panels B, D). Both approaches consistently identified the same root for each subgroup cluster.

Comprehensive characterization of the datasets was achieved through phylodynamic analysis. The most recent common ancestor (MRCA) was dated to 1951 for HRSV-A and 1965 for HRSV-B (Appendix Figure 3, panels E,F). Global evolutionary rates were estimated to $7.964 \times 10^{-4}$ substitutions/site/year ($r^2 = 0.96$) for HRSV-A and $6.933 \times 10^{-4}$ substitutions/site/year ($r^2 = 0.98$) for HRSV-B, aligning with previous reports (*10–12*).

Outlier sequences in root-to-tip plots evaluating the temporal signal may be unreliable due to, for example, sequencing errors and/or inaccurate metadata. We identified 58 outlier sequences for HRSV-A and 2 for HRSV-B that were excluded from rates estimation and lineage designation. Following the exclusion of outliers from the root-to-tip analysis, the final dataset considered for lineage designation comprised 1,480 HRSV-A genomes and 1,385 HRSV-B genomes.

### HRSV Lineage Definition

Results of the phylogenetic clustering using from 1 to 10 amino acids as thresholds in an automated manner are available at https://github.com/rsv-lineages/Classification_proposal.

### Use of G and F Sequences with the HRSV Lineage Classification System

We assessed the reproducibility of the classification with the G and/or F gene sequences. The phylogenetic trees obtained are available at https://github.com/rsv-lineages/Classification_proposal.

Our assessment of using this classification with solely the G gene showed minimal misclassification (1.2% error) in HRSV-A, and none in HRSV-B (Appendix Figure 5). This result underscored strong support for lineage-defining nodes. However, employing the G ectodomain alone led to a misclassification rate of 18.86% in HRSV-A due to the association of A.D.1.3 directly from A.D (outside A.D.1 clade), with no misclassification in HRSV-B.

Conversely, relying solely on the F gene resulted in a misclassification of 38.18% and 1.23% in HRSV-A and HRSV-B sequences, respectively. The high rate of HRSV-A misclassification using solely the F gene is related with the appearance of polytomies that hampered the assignation of descendant lineages within A.D.1 and A.D.5. Interestingly, when using a fragment containing both G and F genes, misclassification dropped to 0.07% in HRSV-A and remained absent in HRSV-B, suggesting that incorporating both genes provides optimal resolution for both HRSV subgroups (Appendix Figure 5).

**Prospective HRSV Lineage Assignment and Definition**

HRSV Lineage Assignment

The assignment of sequences to the existing lineages can be automated using online tools such as NextClade (https://clades.nextstrain.org/) (*13*), ReSVidex (https://cacciabue.shinyapps.io/resvidex/), INSaFLU (https://insaflu.insa.pt) (*14*,*15*) or UShER (https://usher.bio/) (*16*). However, for the classical approach to define the lineage of query sequences we encourage users to follow the guidelines described below:

1. Perform an alignment (for instance, with MAFFT, online version (*17*): https://mafft.cbrc.jp/alignment/server/) with the query sequences and the most recent reference alignment of the same HRSV subgroup available in the GitHub (https://github.com/rsv-lineages/lineage-designation-A and https://github.com/rsv-lineages/lineage-designation-B).
2. Visually verify that the alignment covers the entire genomic region from the first codon of NS1 gene to the codon of the L gene. If the target sequences are longer than the reference alignments, trim the ends accordingly. If the target sequences are shorter, no trimming is necessary.
3. Visually evaluate the alignment, especially around the G gene region, to detect and correct alignment artifacts.
4. Infer a maximum likelihood phylogenetic tree with software such as IQ-TREE enabling the selection of the nucleotide substitution model with ModelFinder and assessing node support with UFBoot2 and SH-alrt. Phylogenetic analysis can be

run online (http://iqtree.cibiv.univie.ac.at/) (*18*), or locally using the command line as follows (version IQ-TREE v2.2.0):

```
iqtree2 -s <path_to_sequences> -alrt 1000 -B 1000
```

5. Visualize the phylogenetic tree with software such as FigTree. Ensure that the tree is properly rooted against A.1 or B.1 lineage clades. Find the lineage of the query sequences by associating them with the reference sequences. Evaluate whether the query sequences form a monophyletic clade with statistical support (≥90% for UFBoot2 and ≥80% for SH-alrt).

If, for example, one exclusively uses G gene sequences, the lineage assignment will be based in phylogenetic association. Although all lineages contain amino acid markers in the G gene, there are amino acid- defining lineages in other genes that will not be able to be detected. When using the G gene sequence for lineage assignment, it is recommended to trim the reference alignment from GitHub to the longest G open reading frame and verify the alignment including the query sequences mainly around the duplication region. Interpretating the results when complete genomes are not used should take into consideration the intrinsic limitations.

Inadequate sequencing depth often leads to missing data in genetic sequences, typically denoted by the letter 'N'. This missing data within a sequence may impact the accuracy of its phylogenetic clade association since in maximum likelihood trees constructed with IQ-TREE, missing characters are treated similarly to nucleotide gaps (http://www.iqtree.org/doc/Frequently-Asked-Questions). Consequently, the estimation of nucleotide site-likelihood relies on the data contained in the sequences with non-gap/missing data. However, nucleotide ambiguities, which represent multiple possible bases (e.g., R to represent A or G -purine-, Y to represent C or T -pyrimidine-), are supported in a manner that all represented bases are considered to have equal likelihood. It is crucial to note that sequences containing nucleotide ambiguities, whether they are missing data (N) or represent more than one base (such as R and Y), are still used for phylogenetic classification and identification of the HRSV lineage. However, the presence of any nucleotide ambiguities can hinder the identification of a given lineage-defining amino acid. Therefore, the definition of novel lineages requires complete genomes without any nucleotide ambiguities.

HRSV Novel Lineage Definition

We anticipate that new lineages of HRSV-A and HRSV-B will continue to emerge in future and envision our proposed nomenclature being expanded to incorporate new lineages. The detection and definition of a new lineage comprise the use of complete genomes that should adhere to the considerations outlined in this study on the phylogenetic and amino acid criteria. The recommended procedure to define a new lineage is described below:

1. Perform an alignment (for instance, with MAFFT, online version (*17*): https://mafft.cbrc.jp/alignment/server/) of the complete genomes of the potential new lineage with the reference alignment available on GitHub (https://github.com/rsv-lineages/lineage-designation-A and https://github.com/rsv-lineages/lineage-designation-B).
2. Ensure that complete genomes cover information from the first codon of the first gene (NS1) to the last codon of the last gene (L) without the presence of ambiguous nucleotides. Trim the alignment if necessary to meet the defined criteria for complete genomes. Conversely, sequences can include missing data only in the first or last 50 nt of the alignment. Visually inspect the alignment to identify and correct bioinformatics artifacts, particularly in the G gene region.
3. Infer a maximum likelihood phylogenetic tree with IQTREE, enabling the selection of the nucleotide substitution model using ModelFinder, and assessing statistical support for nodes with UFBoot2 and SH-alrt. Phylogenetic analysis can be run online (http://iqtree.cibiv.univie.ac.at/) (*18*), or by the command line as follows (IQ-TREE v2.2.0):

```
iqtree2 -s <path_to_sequences> -alrt 1000 -B 1000
```

4. Evaluate the phylogenetic tree (in IQTREE v2.2.0, the file with a ".treefile" extension) to confirm that the potential new lineage meets the phylogenetic lineage criteria. This entails a monophyletic clade with at least 10 sequences of interest, supported by statistical values ≥90% for UFBoot2 and ≥80% for SH-alrt. The potential lineage should not contain sequences from any other lineage.

5. Conduct a comparative analysis of the protein sequences of the potential lineage and its parental lineage to confirm the amino acid lineage criteria. Subset the complete genomes alignment into individual open reading frames and translate the nucleotides using the universal genetic code. Identify among all the viral proteins at least 5 aa substitutions differentiating the potential lineage from the parental one. Those amino acids should present in more than 90% of the new lineage sequences.

6. If the potential lineage meets both the phylogenetic and amino acid substitution requirements, it can be named following the lineage nomenclature, including the suffix “x” to denote its potential status. For example, A.D.1.3.1pot for a descendant of A.D.1.3 and B.D.E.2.1x for a descendant of B.D.E.2. Another example based on the current state is A.D.E.1x for a descendant of A.D.2.2.1, where E is the alias for 2.2.1.

7. Share the proposal of the new lineage on the RGCC GitHub page as an 'issue' within the corresponding repository for HRSV-A (https://github.com/rsv-lineages/lineage-designation-A) or HRSV-B (https://github.com/rsv-lineages/lineage-designation-B). The RGCC study group will evaluate the new proposed lineage, and if accepted, the reference alignments will be updated.

**References**


1. Minh BQ, Schmidt HA, Chernomor O, Schrempf D, Woodhams MD, von Haeseler A, et al. IQ-TREE 2: New Models and Efficient Methods for Phylogenetic Inference in the Genomic Era. Mol Biol Evol. 2020;37:1530–4. PubMed https://doi.org/10.1093/molbev/msaa015

2. Kalyaanamoorthy S, Minh BQ, Wong TKF, von Haeseler A, Jermiin LS. ModelFinder: fast model selection for accurate phylogenetic estimates. Nat Methods. 2017;14:587–9. PubMed https://doi.org/10.1038/nmeth.4285

3. Hoang DT, Chernomor O, von Haeseler A, Minh BQ, Vinh LS. UFBoot2: Improving the Ultrafast Bootstrap Approximation. Mol Biol Evol. 2018;35:518–22. PubMed https://doi.org/10.1093/molbev/msx281

4. NextStrain. auspice.us. https://auspice.us/

5. Rambaut A. Figtree. 2023. https://github.com/rambaut/figtree

6. Sagulenko P, Puller V, Neher RA. TreeTime: Maximum-likelihood phylodynamic analysis. Virus Evol. 2018;4:vex042. PubMed https://doi.org/10.1093/ve/vex042

7. Rambaut A, Lam TT, Max Carvalho L, Pybus OG. Exploring the temporal structure of heterochronous sequences using TempEst (formerly Path-O-Gen). Virus Evol. 2016;2:vew007. PubMed https://doi.org/10.1093/ve/vew007

8. Martin DP, Murrell B, Golden M, Khoosal A, Muhire B. RDP4: Detection and analysis of recombination patterns in virus genomes. Virus Evol. 2015;1:vev003. PubMed https://doi.org/10.1093/ve/vev003

9. Barrat-Charlaix P, Vaughan TG, Neher RA. TreeKnit: Inferring ancestral reassortment graphs of influenza viruses. PLOS Comput Biol. 2022;18:e1010394. PubMed https://doi.org/10.1371/journal.pcbi.1010394

10. Goya S, Lucion MF, Shilts MH, Juárez MDV, Gentile A, Mistchenko AS, et al. Evolutionary dynamics of respiratory syncytial virus in Buenos Aires: viral diversity, migration, and subgroup replacement. Virus Evol. 2023;9:vead006.

11. Tan L, Lemey P, Houspie L, Viveen MC, Jansen NJG, van Loon AM, et al. Genetic variability among complete human respiratory syncytial virus subgroup A genomes: bridging molecular evolutionary dynamics and epidemiology. PLoS One. 2012;7:e51439. PubMed https://doi.org/10.1371/journal.pone.0051439

12. Di Giallonardo F, Kok J, Fernandez M, Carter I, Geoghegan JL, Dwyer DE, et al. Evolution of human respiratory syncytial virus (RSV) over multiple seasons in New South Wales, Australia. Viruses. 2018;10:476. PubMed https://doi.org/10.3390/v10090476

13. Aksamentov I, Roemer C, Hodcroft EB, Neher RA. Nextclade: clade assignment, mutation calling and quality control for viral genomes. J Open Source Softw. 2021;6:3773. https://doi.org/10.21105/joss.03773

14. Borges V, Pinheiro M, Pechirra P, Guiomar R, Gomes JP. INSaFLU: an automated open web-based bioinformatics suite "from-reads" for influenza whole-genome-sequencing-based surveillance. Genome Med. 2018;10:46. PubMed https://doi.org/10.1186/s13073-018-0555-0

15. INSaFLU-TELEVIR: an open web-based bioinformatics suite for viral metagenomic detection and routine genomic surveillance. 2023. https://www.researchsquare.com

16. Turakhia Y, Thornlow B, Hinrichs AS, De Maio N, Gozashti L, Lanfear R, et al. Ultrafast Sample placement on Existing tRees (UShER) enables real-time phylogenetics for the SARS-CoV-2 pandemic. Nat Genet. 2021;53:809–16. PubMed https://doi.org/10.1038/s41588-021-00862-7

17. Rozewicki J, Li S, Amada KM, Standley DM, Katoh K. MAFFT-DASH: integrated protein sequence and structural alignment. Nucleic Acids Res. 2019;47(W1):W5–10. PubMed https://doi.org/10.1093/nar/gkz342

18. Trifinopoulos J, Nguyen LT, von Haeseler A, Minh BQ. W-IQ-TREE: a fast online phylogenetic tool for maximum likelihood analysis. Nucleic Acids Res. 2016;44(W1):W232-5. PubMed https://doi.org/10.1093/nar/gkw256

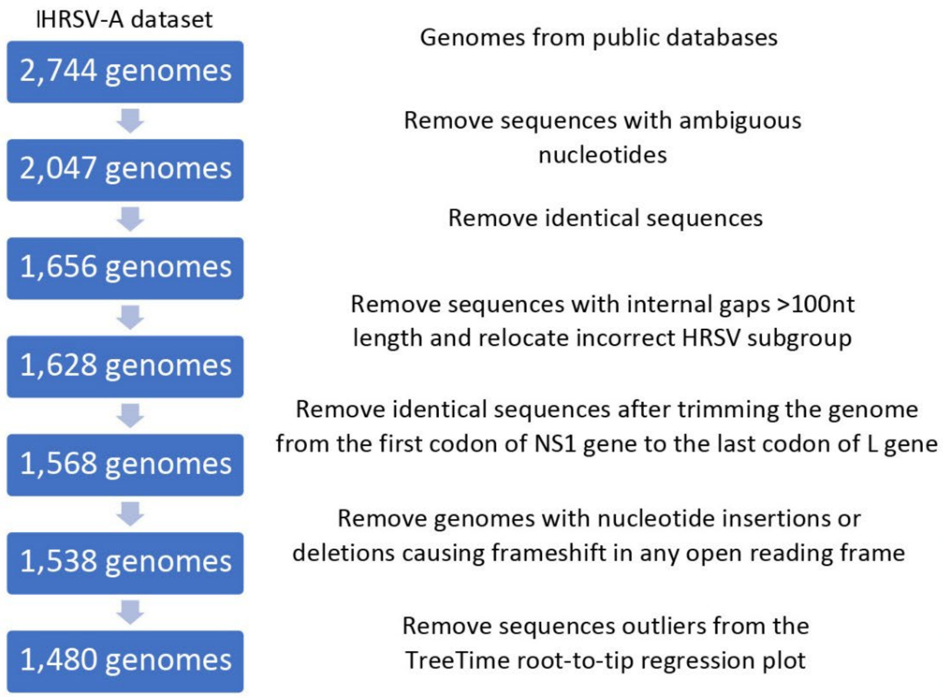


**Appendix Figure 1.** Dataset curation for the HRSV classification definition. For each of the filtration step during the dataset curation the number of remaining HRSV-A and HRSV-B genomes is detailed.

| RSV genomes | Year | Argentina | Brazil | Canada | Nicaragua | USA | Cote d'Ivoire | Egypt | Kenya | Morocco | South Africa | Uganda | Cambodia | China | India | Israel | Japan | Jordan | Lebanon | Philippines | Russia | Thailand | Vietnam | Australia | New Zealand | Austria | Belgium | Bosnia and Herzegovina | France | Germany | Macedonia | Netherlands | Spain | Switzerland | United Kingdom |
|---|---|---|---|---|---|---|---|---|---|---|---|---|---|---|---|---|---|---|---|---|---|---|---|---|---|---|---|---|---|---|---|---|---|---|---|
| 1 | 1956 | | | | | 1 | | | | | | | | | | | | | | | | | | | | | | | | | | | | | |
| 1 | 1979 | | | | | 1 | | | | | | | | | | | | | | | | | | | | | | | | | | | | | |
| 8 | 1980 | | | | | 8 | | | | | | | | | | | | | | | | | | | | | | | | | | | | | |
| 2 | 1981 | | | | | 2 | | | | | | | | | | | | | | | | | | | | | | | | | | | | | |
| 9 | 1982 | | | | | 9 | | | | | | | | | | | | | | | | | | | | | | | | | | | | | |
| 1 | 1983 | | | | | 1 | | | | | | | | | | | | | | | | | | | | | | | | | | | | | |
| 4 | 1984 | | | | | 4 | | | | | | | | | | | | | | | | | | | | | | | | | | | | | |
| 5 | 1985 | | | | | 5 | | | | | | | | | | | | | | | | | | | | | | | | | | | | | |
| 3 | 1986 | | | | | 3 | | | | | | | | | | | | | | | | | | | | | | | | | | | | | |
| 5 | 1987 | | | | | 5 | | | | | | | | | | | | | | | | | | | | | | | | | | | | | |
| 1 | 1988 | | | | | 1 | | | | | | | | | | | | | | | | | | | | | | | | | | | | | |
| 2 | 1989 | | | | | 2 | | | | | | | | | | | | | | | | | | | | | | | | | | | | | |
| 3 | 1990 | | | | | 3 | | | | | | | | | | | | | | | | | | | | | | | | | | | | | |
| 4 | 1991 | | | | | 4 | | | | | | | | | | | | | | | | | | | | | | | | | | | | | |
| 2 | 1992 | | | | | 2 | | | | | | | | | | | | | | | | | | | | | | | | | | | | | |
| 2 | 1993 | | | | | 2 | | | | | | | | | | | | | | | | | | | | | | | | | | | | | |
| 13 | 1994 | | | | | 13 | | | | | | | | | | | | | | | | | | | | | | | | | | | | | |
| 13 | 1995 | | | 4 | | 9 | | | | | | | | | | | | | | | | | | | | | | | | | | | | | |
| 1 | 1996 | | | | | 1 | | | | | | | | | | | | | | | | | | | | | | | | | | | | | |
| 20 | 2001 | | | | | 20 | | | | | | | | | | | | | | | | | | | | | | | | | | | | | |
| 9 | 2002 | | | | | 9 | | | | | | | | | | | | | | | | | | | | | | | | | | | | | |
| 15 | 2003 | | | | | 15 | | | | | | | | | | | | | | | | | | | | | | | | | | | | | |
| 5 | 2004 | | | | | 5 | | | | | | | | | | | | | | | | | | | | | | | | | | | | | |
| 4 | 2005 | | | | | 4 | | | | | | | | | | | | | | | | | | | | | | | | | | | | | |
| 7 | 2007 | | | | | | | | 7 | | | | | | | | | | | | | | | | | | | | | | | | | | |
| 50 | 2008 | | 43 | | | 1 | | | 1 | | | | | | | | | | | | | | | | | | | | | | | | | | 5 |
| 55 | 2009 | | 38 | | | 1 | | | 1 | | | | | 1 | | | | | | | | | | | | | | | | | | | | | 14 |
| 99 | 2010 | | 31 | | | | | | 51 | | | | | | | | | 3 | | | | | | | | | | | | | | | | | 14 |
| 22 | 2011 | | | | | | | | 2 | | | | | | | | | 12 | | | | 1 | | | | | | | | | | | | | 7 |
| 114 | 2012 | | | | | 3 | | | 67 | 7 | | | | 1 | | | | 2 | | 7 | | 1 | | | | | | | | | | | | | 26 |
| 58 | 2013 | | | | 1 | 4 | | | 16 | | | | | 1 | | | | 10 | | 6 | | 2 | | | | | | | | | | | | | 18 |
| 112 | 2014 | 53 | | | | 2 | | | 17 | 13 | | | | 4 | | | | | 1 | 4 | | 2 | | | | | | | | | | | | | 16 |
| 141 | 2015 | 56 | | | 27 | 14 | | | 17 | 13 | | | | | | | | | | 2 | | | | 2 | | | | | | 2 | | | | | 8 |
| 161 | 2016 | 48 | | | 24 | 13 | | | 4 | 48 | | | | | | | 1 | | | 2 | | 2 | | 1 | | | | | | | | | | | 18 |
| 134 | 2017 | 47 | | | | 1 | 6 | | | 40 | | | | 2 | 1 | | | | | | | 2 | 2 | 2 | | | | | | | | 22 | 3 | | 6 |
| 164 | 2018 | 17 | | | | 1 | 2 | | | 5 | | | | 4 | | | | | | | 2 | | 2 | 4 | | | | | | | | 48 | 10 | | 69 |
| 215 | 2019 | 5 | 1 | | | 9 | 4 | 2 | | 15 | | | | | | | | | | 24 | 9 | | | 4 | | 2 | | | | | | 53 | 31 | 1 | 55 |
| 196 | 2020 | | | | | 2 | | 3 | | | | | | 17 | | | | | | 4 | 4 | | 2 | 140 | | 1 | | | | | | 2 | 11 | | 10 |
| 741 | 2021 | 8 | 1 | | | 6 | | | | | 58 | 1 | 1 | 19 | | 2 | | | | | 1 | | | 182 | 390 | 1 | | | 8 | | | | | | 63 |
| 463 | 2022 | | 1 | | | 72 | | | | | 33 | | | | | | | | | | | | | 250 | | 3 | 1 | 1 | | | 4 | | | | 98 |

**Appendix Figure 2.** Characterization of the genomes used in the classification. The number of HRSV genomes per year and the country of sample collection are reported. Countries are grouped into geographic regions highlighted in gray scale. The heat map highlights the countries representation by collection year.

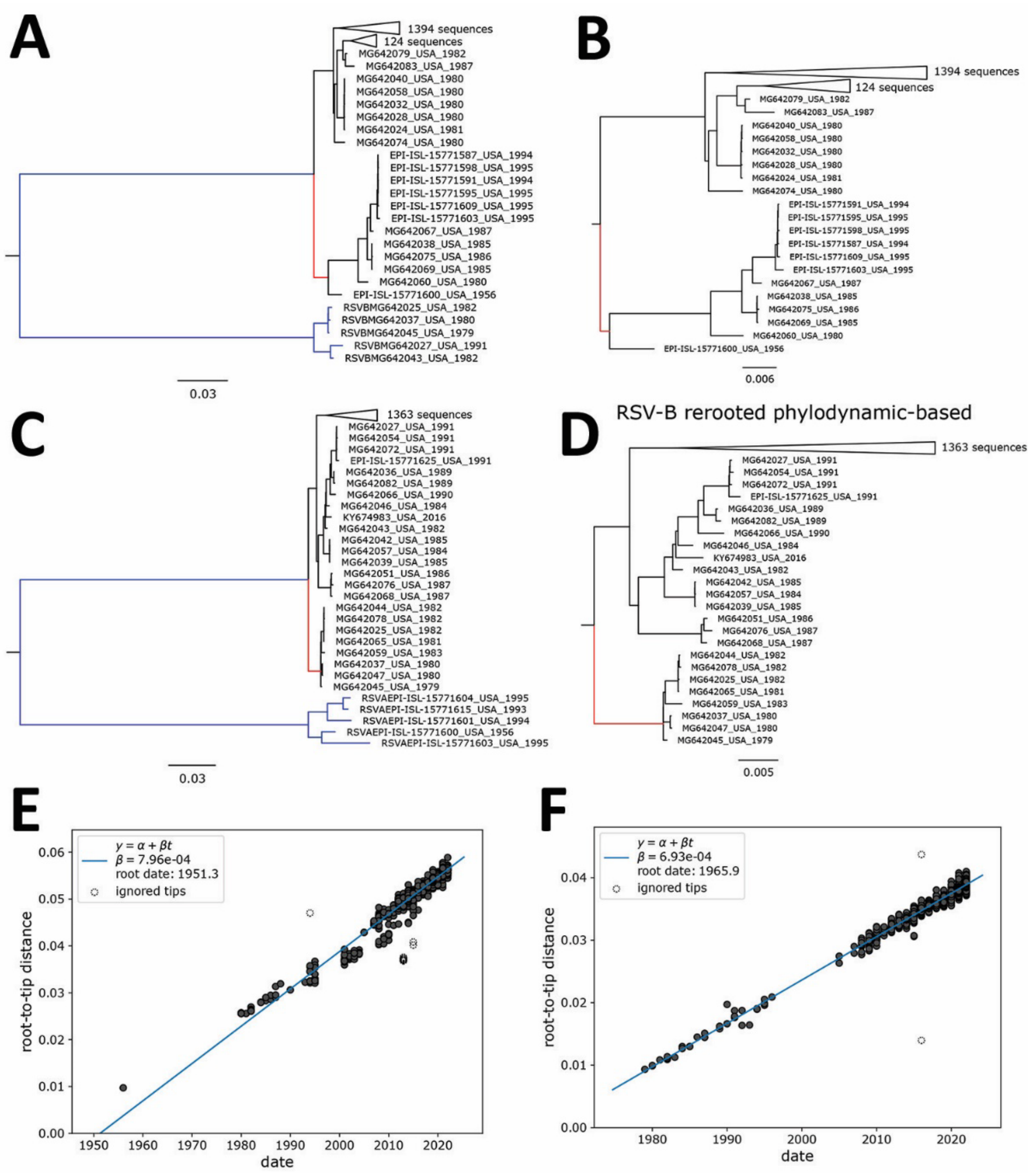


**Appendix Figure 3.** Rooting the HRSV phylogenetic trees. (A-B) HRSV-A maximum-likelihood (ML) phylogenetic tree rooted using an outgroup of HRSV-B sequences highlighted in blue is shown as well as the tree rooted by phylodynamic analysis. (C-D) Similarly in HRSV-B, the ML phylogenetic tree rooted using an outgroup of HRSV-A highlighted in blue and the tree rooted by phylodynamic analysis are shown. The branch indicating the common ancestor of the HRSV-A or HRSV-B sequences is indicated in red. (E-F) The genetic distances from the root to the tips of the ML tree are plotted against the year of collection. Root-to-tip mutation counts vs. sample collection date for HRSV-A and HRSV-B. Regression was plotted using to the best fitting root which minimizes the sum of the squared residuals from the regression line. The x-intercept of the regression line represents the estimated time of the most recent common ancestor of the dataset in the tree, while the gradient estimates the evolutionary rate. Outliers excluded from rate estimation are indicated.

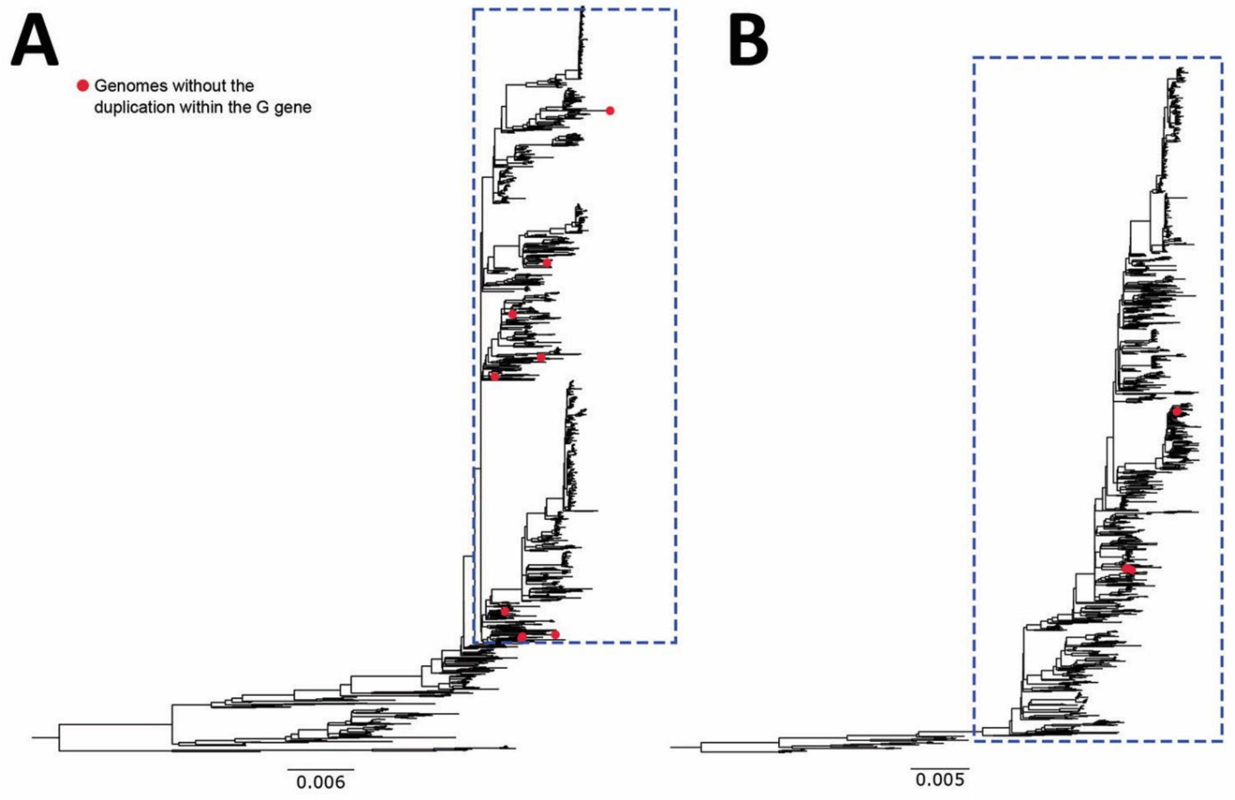


**Appendix Figure 4.** Allocation of HRSV-A and HRSV-B without the duplication in the G gene. Genomes lacking the duplication of G gene in the dataset for the classification definition that were found within the clade containing the duplication of G are denoted with a red circle in the HRSV-A or HRSV-B maximum likelihood tree. The clade of A.D. or B.D. and nested lineages in HRSV-A or HRSV-B, respectively is denoted with a blue dashed line box.

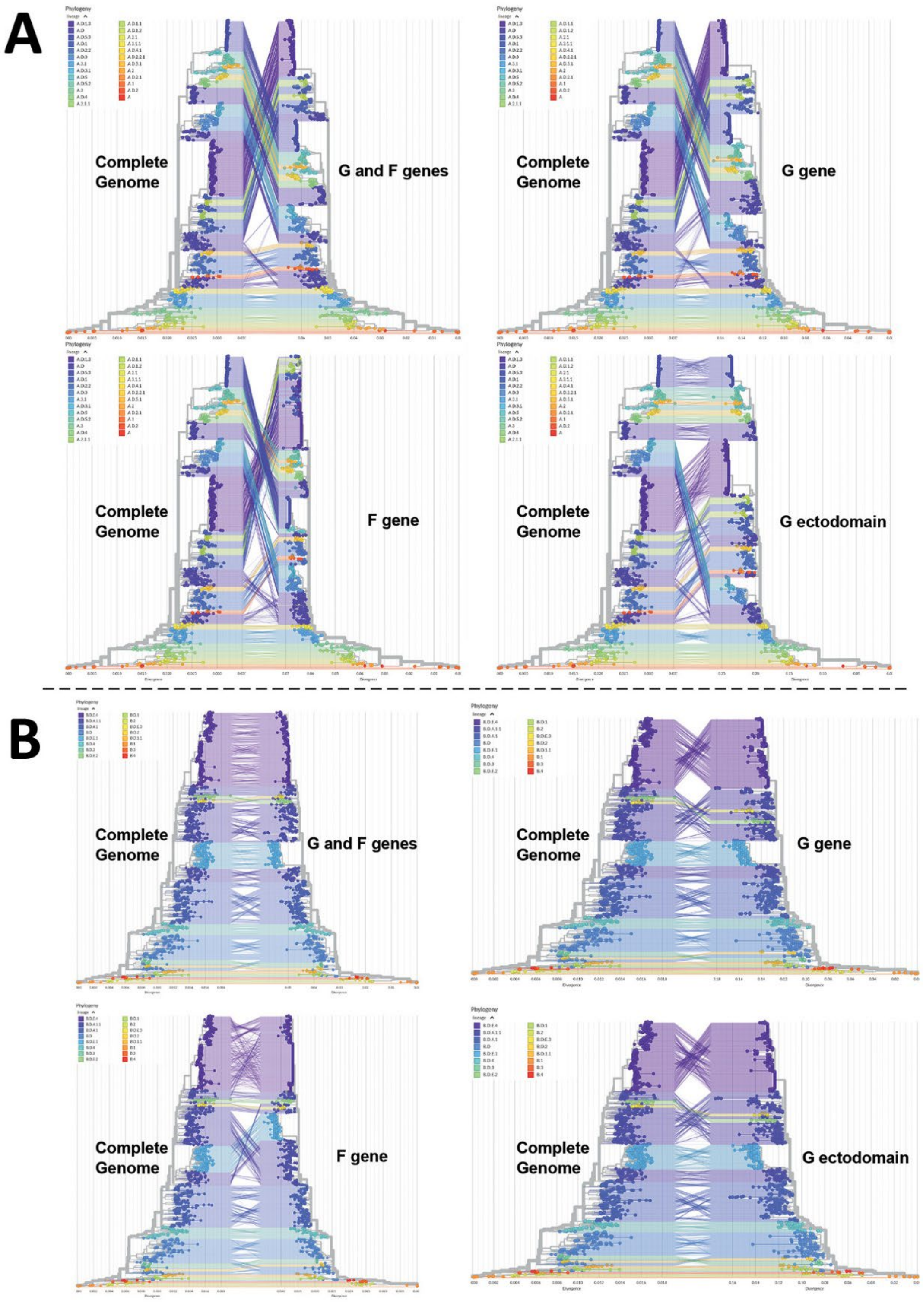


**Appendix Figure 5.** Tanglegrams of HRSV-A and HRSV-B maximum likelihood trees constructed using complete genomes, genomic fragment comprising G and F genes, the G and F genes independently, and the G ectodomain region. The positions of the sequences in both phylogenetic trees are highlighted according to their lineage classification.

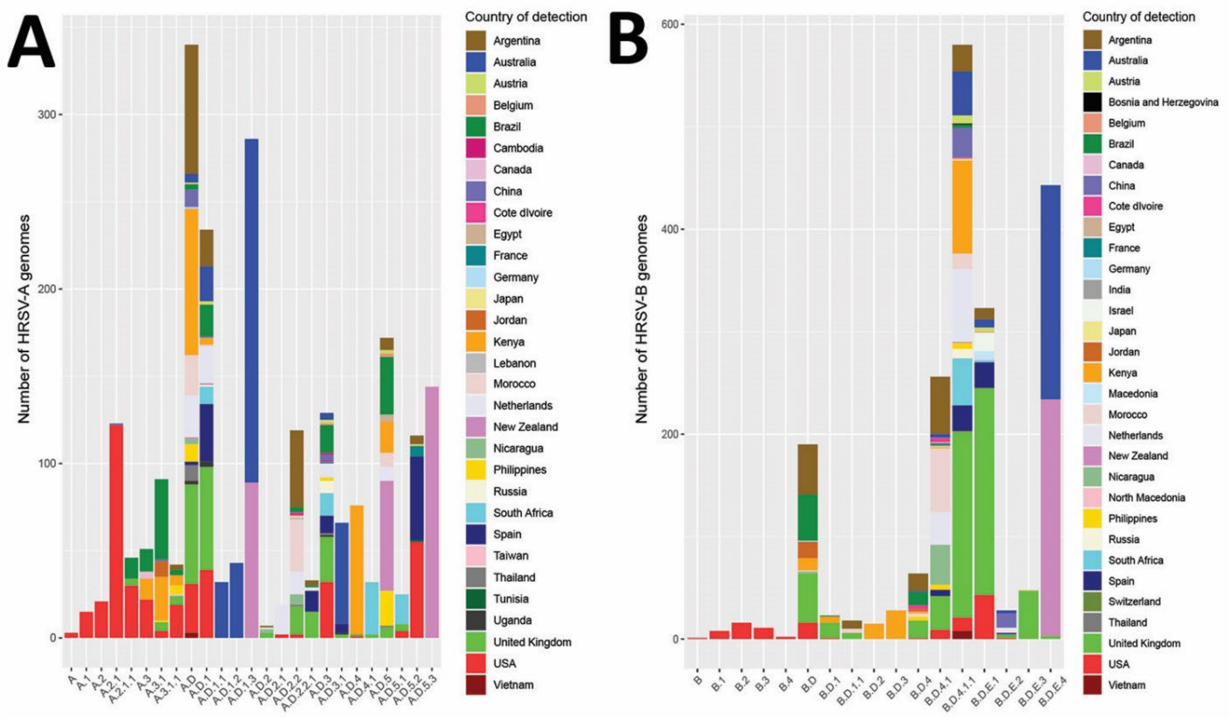


**Appendix Figure 6.** Geo-detection of HRSV-A and HRSV-B lineages. For each lineage, the number of genomes detected per country is reported in the stacked bar plots.
